# Changes in selection of resources with reproductive state in a montane ungulate

**DOI:** 10.1186/s40462-023-00378-1

**Published:** 2023-04-05

**Authors:** Marcus E. Blum, Kelley M. Stewart, Kevin T. Shoemaker, Mike Cox, Brian F. Wakeling, Thomas E. Dilts, Joe R. Bennett, Vernon C. Bleich

**Affiliations:** 1 Natural Resources Institute, Texas A&M University, 1001 Holleman Dr, College Station, TX 77840 USA; 2 Department of Natural Resources and Environmental Science, University of Nevada, Reno, 1664 N. Virginia St., MS 186, Reno, NV 89557 USA; 3 Nevada Department of Wildlife, 6980 Sierra Center Parkway #120, Reno, NV 89511 USA; 4 Montana Fish, Wildlife, and Parks, P.O. Box 200701, Helena, MT 59620 USA

**Keywords:** Bayesian, Desert bighorn sheep, *Ovis canadensis nelsoni*, Reproduction, Resource selection, Risk-averse, Risk-prone, Tradeoff

## Abstract

Animals select habitats based on food, water, space, and cover. Each of those components are essential to the ability of an individual to survive and reproduce in a particular habitat. Selection of resources is linked to reproductive fitness and individuals likely vary in how they select resources relative to their reproductive state: during pregnancy, while provisioning young when nutritional needs of the mother are high, but offspring are vulnerable to predation, or if they lose young to mortality. We investigated the effects of reproductive state on selection of resources by maternal female desert bighorn sheep (*Ovis canadensis nelsoni*) by comparing selection during the last trimester of gestation, following parturition when females were provisioning dependent young, and if the female lost an offspring. We captured, and recaptured each year, 32 female bighorn sheep at Lone Mountain, Nevada, during 2016–2018. Captured females were fit with GPS collars and those that were pregnant received vaginal implant transmitters. We used a Bayesian approach to estimate differences in selection between females provisioning and not provisioning offspring, as well as the length of time it took for females with offspring to return levels of selection similar to that observed prior to parturition. Females that were not provisioning offspring selected areas with higher risk of predation, but greater nutritional resources than those that were provisioning dependent young. When females were provisioning young immediately following parturition, females selected areas that were safe from predators, but had lower nutritional resources. Females displayed varying rates of return to selection strategies associated with access to nutritional resources as young grew and became more agile and less dependent on mothers. We observed clear and substantial shifts in selection of resources associated with reproductive state, and females exhibited tradeoffs in favor of areas that were safer from predators when provisioning dependent young despite loss of nutritional resources to support lactation. As young grew and became less vulnerable to predators, females returned to levels of selection that provided access to nutritional resources to restore somatic reserves lost during lactation.

**Supplementary Information:**

The online version contains supplementary material available at 10.1186/s40462-023-00378-1.

## Background

Animals select habitats based on food, water, and cover, and each of those is essential to the ability of an individual to survive and reproduce in a particular habitat. Therefore, understanding how individuals select resources that they need to survive, reproduce, and recruit young provides insight into how selection of resources enhances reproductive fitness [1–3]. Measuring variation in selection of habitats and how selection varies across reproductive states of individuals is critically important for understanding how individuals interact with the ecosystems they inhabit, which may influence how populations are managed and improve conservation efforts of target species [4–7]. Failure to account for this variation may result in conservation strategies that do not improve a species’ habitat requirements for various life history strategies, thus reducing reproductive fitness within populations. Life-history characteristics of animals drive demographic processes and selection of resources, and, therefore, directly influence fitness of individuals [3, 8, 9]. Animals that exhibit slow-paced life histories, such as ungulates, invest heavily in fewer offspring than those with fast-paced life histories [10]. Long-lived species, with slow-paced life histories, sometimes make tradeoffs between current and future reproduction [11–16]. For instance, Morano et al. [15] reported a negative relationship between recruitment in the current year and pregnancy rates of North American elk (*Cervus canadensis*) the following year, indicating that females were trading the uncertainties of future reproduction for assurance of successful reproduction in the current year. Additionally, slow-paced species may be forced to make tradeoffs in how they select resources based on specific stages of reproduction. Females likely change how they select resources when they are pregnant, provisioning dependent young, or following mortality of an offspring [17, 18]. In addition to changing selection of resources by reproductive state, changes in selection would be expected to occur with varying nutritional needs of mothers (i.e., costs of lactation reduce as the neonate ages and requires less milk) to successfully provision those young, but those needs must be balanced with the need to provide for safety of neonatal offspring from predators.

Given that selection of habitats or resources by maternal females likely varies as a function of reproductive state, females may select safer or less risky habitats when provisioning neonatal young, compared with pregnancy or following mortality of offspring [18–22]. Immediately following parturition, females may forego access to nutritional resources in favor of safety of young by selecting areas with lower risk of predation [23–26]. Nevertheless, those habitats that provide security for dependent young have lower risk of predation, but generally do not provide adequate nutritional resources for lactating females. Habitats selected by females that are safer for neonatal offspring but have lower quality forages to support maternal nutritional needs are described as being “risk-averse” [19, 20, 27]. Habitats with greater nutritional resources are commonly described as more risky because, in many instances, those habitats with higher-quality forage often present a greater risk of predation [19, 20, 27, 28]. Females that are pregnant or have lost their young would likely be focused on obtaining nutritional resources to support themselves for use during lactation or to replenish lost somatic resources following loss of their offspring [18]. Thus, behaviors of maternal females when selecting habitats to support their own nutrition needs, albeit with potentially higher risk of predation on young, are described as being “risk-prone” [19, 20, 27].

Large herbivores that inhabit montane habitats have been documented to exhibit tradeoffs in selection of habitats that provide safety for offspring (risk-averse behavior), or to support nutritional needs of maternal females (risk-prone behavior) during reproduction, especially when provisioning dependent young [4, 19, 20, 25, 26, 28]. Those strategies and associated tradeoffs are unlikely to remain static while provisioning offspring, since young generally become more adept at escaping predators as they grow [29–32]. Assuming that offspring are less susceptible to predation as they grow and become less dependent on their mothers, females likely adjust selection of resources to enhance their nutrient intake to replenish somatic reserves depleted during pregnancy and lactation [33, 34]. Those offspring that are recruited into the breeding population may select similar habitat types and exhibit similar selection of resources during reproduction that they learned from their mothers [27, 35–37]. That behavioral strategy may not hold across species or habitats, however, because some habitats may exist in which females can maximize survival of young and nutrient intake simultaneously or experience similar rates of predation and quality of resources across their home range [17, 38].

Mountain sheep (*Ovis* spp.) have a circumpolar distribution, are sexually dimorphic, and are commonly associated with precipitous terrain and climatically harsh landscapes [20, 39]. Additionally, males and females spend most of the year sexually segregated [20] to meet metabolic needs that differ between males and females [40, 41]. As a result, mountain sheep are ideal species to investigate tradeoffs in selection of resources during reproductive periods, because montane landscapes that they inhabit do not often offer habitat types that meet both needs for safety of young and nutrition of mothers simultaneously. Tradeoffs in habitat use or selection of resources between survival of young and nutrition of mothers are common among these specialized artiodactyls [18, 20, 28, 42–44]. Given their wide distribution and variation in use of vegetation types, mountain ungulates are uniquely suited for investigating such tradeoffs.

Female mountain sheep typically select precipitous terrain, which is usually associated with lower availability of nutritional resources for the mother, during parturition or when provisioning dependent young to enhance the probability of offspring survival [20, 24, 25, 39, 45, 46]. While these potential tradeoffs are well documented, the extent and variation of the tradeoffs among differing reproductive states and habitat types are not as well understood. For instance, previous research has not included collared neonates to confirm provisioning status of females and, instead, relied on estimating neonate age through visual observations [20, 24, 25, 45]. Moreover, to our knowledge, no investigators have documented how maternal bighorn sheep shift selection of resources by reproductive state or as offspring grow. Furthermore, it is not understood how females adjust selection of resources, or if tradeoffs cease following the loss of a neonate. Therefore, our understanding of how long these tradeoffs may persist and to what degree has remained elusive. This information is, however, critically important to understanding the nature of tradeoffs in large mammals and how females balance survival of offspring with their own metabolic needs.

**Fig. 1:**
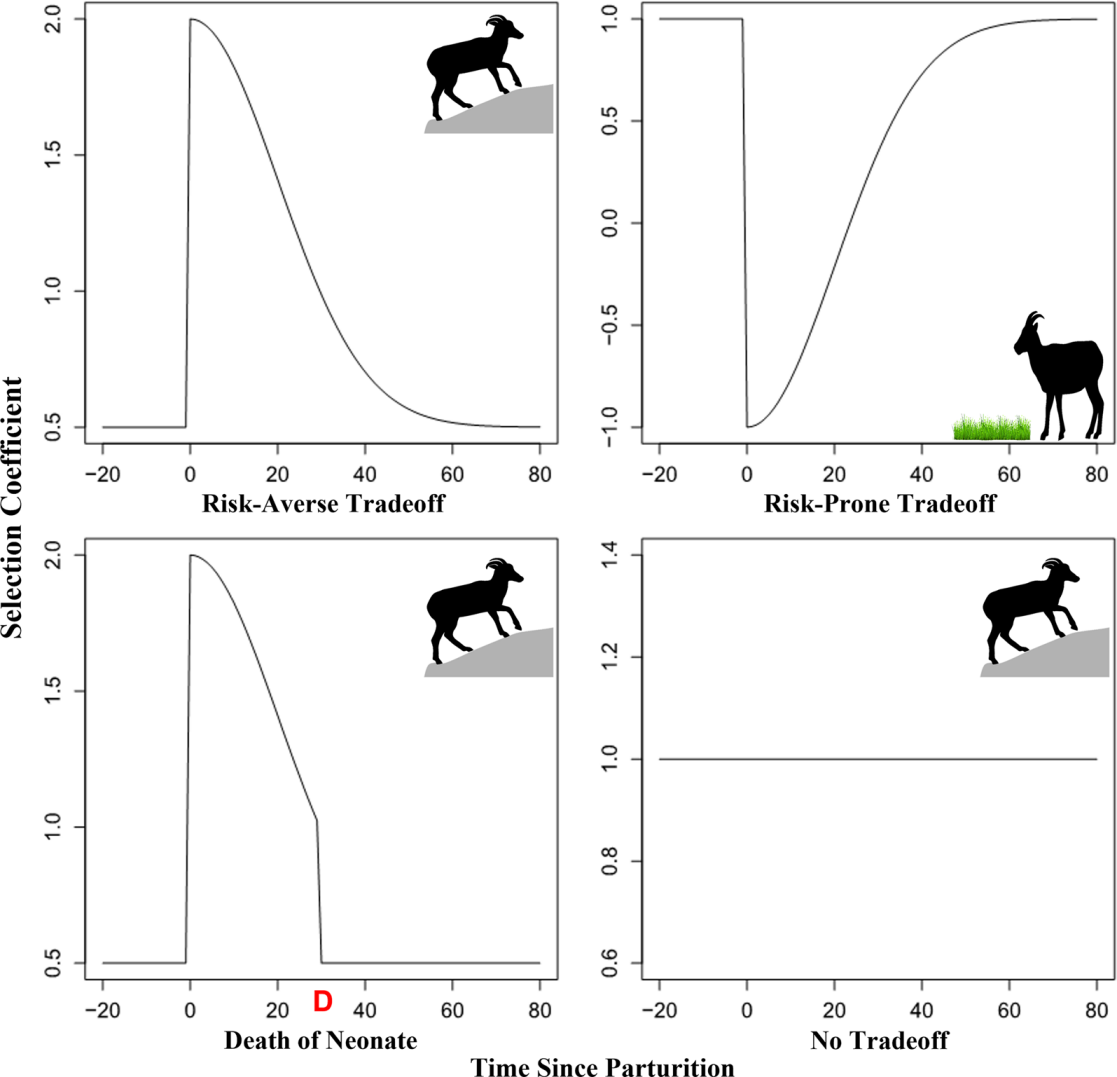
Conceptual model representing potential tradeoffs and variation in habitat selection of desert bighorn sheep associated with reproduction. The top-left pane shows the expected relationship with resources selected by females exhibiting risk averse selection (e.g., slope and terrain ruggedness), where we would expect a shift to higher values of these resources following parturition in the presence of a tradeoff. The top-right pane shows the expected relationship with resources associated with risk prone selection (e.g., high quality vegetation), where we would expect a shift to lower values of these resources following parturition in the presence of a tradeoff. The bottom-left pane shows the expected relationship following the loss of a neonate, denoted by a red “D”, where a female will revert to selection similar to that exhibited prior to parturition. The bottom-right pane shows the potential relationship with habitat variables if there is no effect of parturition on selection

Desert bighorn sheep (*Ovis canadensis nelsoni*) occupy mountain ranges across much of southwestern North America and are associated with rugged landscapes, which often have limited availability of free water [47, 48]. We studied a population of desert bighorn sheep to examine the tradeoff between maternal nutrition and safety of young in the context of selection of resources exhibited by maternal females. Herein we investigate how selection of resources varies by reproductive state and as offspring grow. Our objectives were to identify the resources and habitats selected by female bighorn sheep (1) that are in the third trimester of gestation, (2) immediately following parturition to identify shifts in selection by maternal females with dependent young, (3) determine if females traded nutrient availability for lower risk of predation on young, (4) determine if females that lost offspring immediately shifted selection of resources to enhance nutrient intake, and (5) identify how these potential tradeoffs change with age and growth of offspring. We predicted that females with dependent young select resources and habitats associated with low risk of predation on young (Fig. 1). We also predicted that pre-parturient females, and those that had lost offspring, selected resources and habitats with higher quality nutrition, less rugged terrain, and shallower slopes compared with females provisioning dependent young (Fig. 1). Further, we predicted that females shift selection from areas associated with safety of offspring to those with higher nutritional resources in about a month following parturition, because of reduced risk of predation to older offspring (Fig. 1).

## Methods

### Study area

**Fig. 2:**
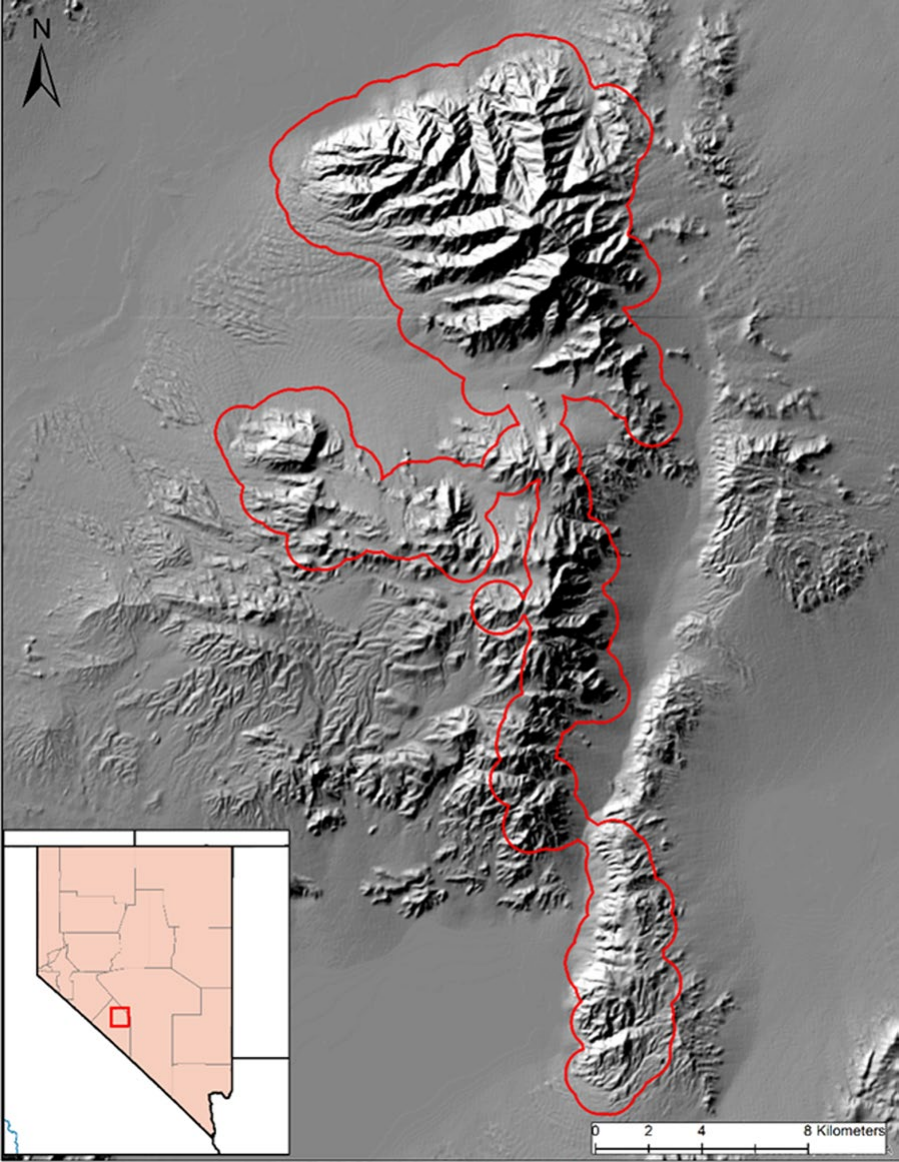
Map of the Lone Mountain study area in west central Nevada. The red boundary in the main figure represents the area defined as available for bighorn sheep (2016–2018). The red square in the inlaid figure shows the study area location within Nevada in addition to the state counties

Our study site was located at Lone Mountain (38^○^ 2′N, 117^○^ 31′W), about 25 km west of Tonopah, Esmeralda Co., Nevada (Fig. 2). Ungulate populations were comprised primarily of desert bighorn sheep and mule deer (*Odocoileus hemionus*), but also included pronghorn (*Antilocapra americana*) and feral horses (*Equus ferus*). The area was host to a suite of predators capable of preying on bighorn sheep, including mountain lion (*Puma concolor*), bobcat (*Lynx rufus*), coyote (*Canis latrans*), grey fox (*Urocyon cinereoargenteus*), and golden eagle (*Aquila chrysaetos*). No effort was made during our study to control or manipulate predator populations. During our study, the bighorn sheep population was considered to be increasing and approaching carrying capacity (unpublished data). Bighorn sheep were distributed across Lone Mountain throughout the year, but portions of the population also occupied the Weepah Hills (37^○^ 56′N, 117^○^ 30′W) outside of summer months, following which they returned to Lone Mountain.

Elevations in the study area ranged from 1400 to 2800 m and supported a variety of plant communities. Lower elevations primarily consisted of vegetation including Wyoming sagebrush (*Artemisia tridentata wyomingensis*), spiny hopsage (*Grayia spinosa*), Stansbury cliffrose (*Purshia stansburiana*), and Nevada ephedra (*Ephedra nevadensis*). Mid- to high-elevations were dominated by Utah Juniper (*Juniperus osteosperma*), singleleaf pinyon pine (*Pinus monophylla*), Stansbury cliffrose, and Wyoming sagebrush. Vegetation at low- to mid-elevations was open, while mid- to high-elevations were dominated by dense pinyon-juniper woodlands. Expansive portions of Lone Mountain were comprised primarily of precipitous terrain, and the Weepah Hills contained only small areas of dense pinyon-juniper woodland. The study area received an average of 11.8 cm (SD = 1.6 cm) of precipitation, and 60.1 cm (SD = 48.2 cm) of snowfall from January 2016 to December 2018. Climate was typical of the Great Basin, and temperatures during summer months ranged from 16.2 °C (SD = 0.9 °C) to 31.1 °C (SD = 0.4 °C) while winter months temperatures ranged from − 4.6 °C (SD = 0.4 °C) to 5.6 °C (SD = 1.4 °C).

### Animal capture and handling

Beginning in January 2016, we captured female desert bighorn sheep (2016: *n* = 16, 2017: *n* = 20, 2018: *n* = 27) on Lone Mountain using a net-gun fired from a helicopter [49, 50] and fit individuals with Iridium Global Positioning System (GPS) satellite collars (VECTRONIC Aerospace). Collars were scheduled to collect sheep locations at a fix rate of 6 locations per day from January to September, to provide efficient sampling intervals for estimating resource selection during gestation, as well as pre-weaning resource selection. Helicopter crews limited capture of individuals to a maximum of 2 females per social group to maintain independence of sampling units. In addition, we inserted vaginal implant transmitters (VITs; VECTRONIC Aerospace) into the vaginal canal of each female identified as pregnant via ultrasonography [51], which allowed us to detect parturition events. We did not consider non-pregnant females in our analyses (2016: *n* = 1, 2017: *n* = 3, 2018: *n* = 5). We recaptured individuals (1 recapture: *n* = 15; 2 recaptures: *n* = 9, 3 recaptures: *n* = 9) in January each subsequent year, through 2018, to acquire multiple years of data from each individual. In the event that collared individuals could not be recaptured, we deployed collars on newly captured individuals (2018: *n* = 6). We measured subcutaneous fat thickness, assigned a body-condition score, and estimated ingesta-free body fat for all individuals [52]. No collared individuals died as a result of capture during our study, but one female aborted her fetus within 3 days of capture and was not included in further analyses.

To investigate if or how adult females changed selection of resources relative to provisioning status, we captured neonates shortly (≥ 4 h after VIT expulsion) following parturition, collected measurements and biological samples, and applied expandable collars to each individual. Neonate collars were set to connect with the dam's satellite collar, through ultra-high frequency signals, and indicated when the neonate was separated from the adult; they also incorporated a mortality sensor to help researchers determine time and cause of death. All handling of animals was approved by the Institutional Animal Care and Use Committee at the University of Nevada, Reno (Protocol #00651) and were within guidelines established by the American Society of Mammalogists for care and handling of wild mammals [53].

### GPS data preparation and analyses

We collected 95,643 GPS locations from collared females from January 2016 to December 2018 and ensured that all collars had a > 90% fix rate (i.e., actual GPS location total collected / total of scheduled GPS locations [54]). In our analyses, we included only females for which we captured neonates and for which we could confirm the survival status of their offspring. To reduce error within our GPS location dataset, we removed all two-dimensional fixes [55]. Additionally, we subset the location dataset to include a maximum of 4 locations per day, two in the morning (between 0400 and 01000) and two in the evening (between 1300 and 2200), to reduce potential for temporal autocorrelation within the dataset.

We created a binary covariate, where 0 indicated a GPS location when a juvenile was not present (i.e., pre-parturition or post mortality) and 1 indicated a location where a juvenile was present (i.e., post parturition through death of juvenile), based on the proximity detection from female-offspring collar linkage. We limited locations that an individual had dependent young to 120 days, by which time juveniles were likely weaned from their mothers [56]. Additionally, we limited locations of individual female sheep that lost a neonate to those that occurred ≤ 30 days following parturition; therefore, females that lost their neonate ≥ 31 days following parturition were not used in our analyses. We generated 2 datasets from those data; the first included pre-parturition and post parturition GPS locations, and the second included GPS locations following the death of a juvenile. We used Arc GIS 10.5 (ESRI, Redlands, California, USA) and program R (4.0.5, R Core Team) to project bighorn sheep locations through weaning and append habitat features to these locations. We used a 10-m resolution digital elevation model (DEM; United States Geological Survey, 2018) to create layers for elevation and slope (degrees). We used locations of perennial water sources and the “ecodist” package in R to create a distance from water layer [57]. We estimated a localized version of the Vector Ruggedness Measure (VRM [58]) to represent terrain ruggedness using methods from Dilts et al. [59] (*In Press)*. We used the Rangeland Analysis Platform (https://rangelands.app; Allred et al. [60]) to define 4 vegetation cover classifications representing annual grasses and forbs, perennial grasses and forbs, shrubs, and trees. All topographic and habitat layers were set to 10-m resolution to minimize variation in spatial resolution for analyses of resource selection.

### Statistical analyses

We defined availability for our habitat selection analyses by buffering all bighorn sheep locations by 780 m, which was the estimated 99% (SD * 2.58) percentile of the distribution of bighorn sheep step lengths. Within the available area, we generated 10 random locations for every GPS location of bighorn sheep. We constructed a resource selection model for reproductive females in which selection coefficients were allowed to vary as a function of daily offspring survival status and days since parturition. Analogous to a conventional RSF, we modeled used and random locations of individual female sheep *i* for each day *j* and observation *k* using a Bernoulli distribution, and estimated the relative probability of using that location, ${p}_{i,j,k}$ as a logit linear function of our hypothesized covariates:

$${\text{logit}}\left( {p_{i,j,k} } \right) = \beta_{0} + \beta_{1,i,j} X_{1,i,j,k} \ldots \beta_{n,i,j} X_{n, i,j,k}$$

We assumed that females prior to parturition would select resources at a constant baseline rate, corresponding to the coefficients in a conventional resource selection model (logistic regression based RSF). Our model allowed coefficients to change abruptly to a new value (either higher or lower than the baseline value) following parturition, and to return to the baseline gradually with time since parturition, following a half-normal distribution with a free parameter (*σ*) representing the rate of return to the baseline (see Fig. 1). The full process model can be expressed as follows:

$$\beta_{n,i,j} = \left( {1 - {\text{Lamb}}_{i,j} } \right) \cdot {\upbeta }_{n\_base} + {\text{Lamb}}_{i,j} \cdot \left[ {\left( {{\upbeta }_{n\_base} + {\upgamma }} \right) \cdot \exp \left( {\frac{{ - {\text{Part}}_{i,j}^{2} }}{{2 \times \sigma^{2} }}} \right)} \right]$$

where $\beta_{n,i,j}$ is the selection coefficient for covariate *n*, adult female *i*, and day *j* post parturition, ${\text{Lamb}}_{i,j}$ is a binary indicator of whether or not a female *i* was previsioning an offspring at time *j*, ${\upbeta }_{n\_base}$ is the baseline selection coefficient for variable *n*, $\upgamma$ is the difference between the base and post parturition levels, ${\mathrm{Part}}_{i,j}$ is the number of days since parturition, and *σ* is the rate of return to the baseline (corresponding to the sigma or standard deviation parameter of the half-normal distribution).

We first implemented this model in a maximum likelihood framework to rank alternative candidate models based on Akaike Information Criterion corrected for small sample sizes (AIC*c*) [61]. We followed an approach similar to Arnold [62] to identify a top model, whereby we removed uninformative parameters (i.e., those that did not improve AIC*c* scores > 2) until we arrived at the top model based on AIC*c* score. We started by including all variables that were not correlated and removed variables to find model combinations that resulted in significantly lower AIC*c* scores (≥ Δ 2 AIC*c*), analogous to backwards stepwise selection. We followed that process until we could no longer improve the top model by removing variables. We then fit the top model in a Bayesian framework using jags [63], which was called from R using “jagsUI” [64].

We used vague prior distributions for all estimated parameters. We estimated the prior distribution for baseline selection and post parturition selection via normal distribution with a mean of 0 and variance of 0.1, while σ was estimated via a uniform distribution with a mean of 1 and variance of 60. Given that σ was from a half-normal distribution, 60 represented a vague prior distribution because doubling this variance equaled the 120 days following parturition events that were present in our model. We ran each model for 20,000 iterations, excluding the first 1,000 iterations for burn-in, on 3 Markov chain Monte Carlo (MCMC) chains. We tested all model iterations for convergence on the joint posterior distribution using Rhat scores [65, 66] and visually assessed trace plots for chain mixture. We reported posterior distributions of each parameter as 0.025 and 0.975 quantiles (i.e., 95% credible intervals). Furthermore, we visualized how selection coefficients changed with time-since-parturition (and associated error) by simulating coefficients (from the Bayesian model) across a sequence of scenarios ranging from pre-parturition through the first 120 days post parturition (assuming neonate survival) and plotting the resulting posterior median and Bayesian credible intervals across this hypothetical.

To determine if resource selection following the loss of a neonate differed from pre- and post-parturition selection, we adjusted the previous model by removing the deterministic equation. Thus, we modeled used and random locations of individual female sheep that lost a neonate ≤ 30 days following parturition for each day and observation using a Bernoulli distribution, and estimated the relative probability of using that location, as a logit linear function of our hypothesized covariates. We included all habitat covariates in this model so, at a minimum, we could compare the pre-parturition and post-mortality selection coefficients. We used the same vague priors that were used for habitat covariates in the previous model.

## Results

Our final dataset contained 20,884 used and 208,840 available locations from 22 collared individuals. Timing of parturition varied slightly by year (2016: $\overline{x }$ = Apr. 22, SD = 18.4 days, *n* = 15; 2017: $\overline{x }$ = Apr. 11, SD = 12 days, *n* = 15; 2018: $\overline{x }$ = Apr. 3, SD = 14.1 days, *n* = 10), but most (28 of 42) parturition events occurred in April. We captured a total of 40 neonates of which all but 1 were from collared females. Neonate survival to 120 days-of-age varied across years (2016: 8 of 15 [53.3%]; 2017: 10 of 15 [66.7%]; 2018: 2 of 10 [20%]). We classified 20 mortalities within the first 120 days of life, including 7 felid predations, 1 eagle predation, 1 neonate that fell in a rock crevice, 1 neonate found dead at the birth site, 3 individuals > 72 h old killed by undetermined predators, and 7 mortalities among individuals ≤ 72 h old that were not the result of predation.

**Fig. 3:**
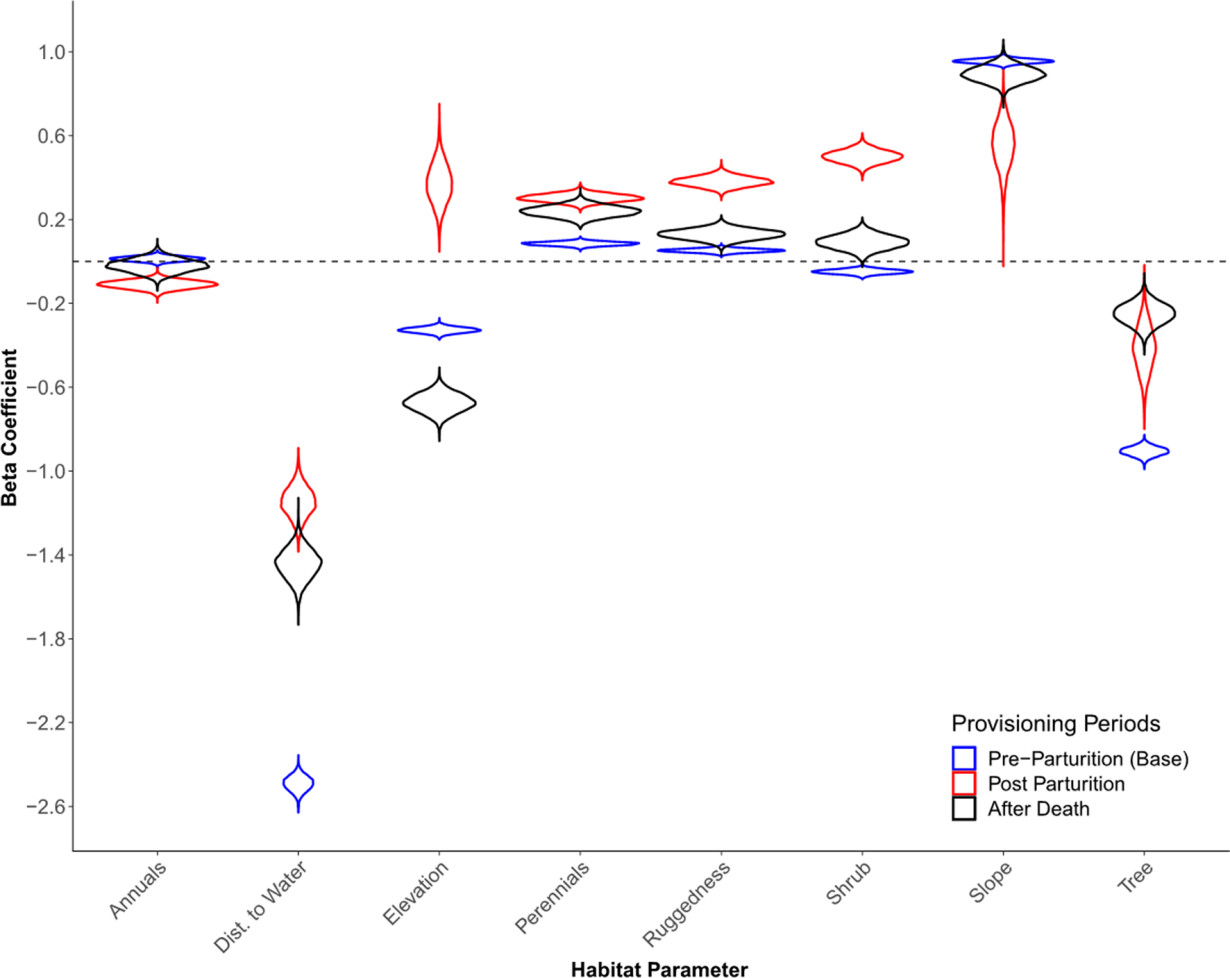
Violin plots of posterior distributions from the top Bayesian model for habitat selection by desert bighorn sheep at Lone Mountain, Nevada (2016–2018). Red violin plots represent those estimated post parturition, blue violin plots represent those estimated prior to parturition (base), and black violin plots represent those estimated following the death of a neonate

Our top model for selection of resources by pre-parturient females indicated differences in selection for all covariates post parturition. Following parturition and while provisioning dependent young, females selected areas that were further from water, in more rugged terrain, on less steep slopes, and at higher elevations than was observed prior to parturition (Table 1, Fig. 3). During this period of provisioning dependent young, females also selected areas with higher shrub cover, higher perennial grass and forb cover, higher tree cover, and lower annual grass and forb cover compared to pre-parturient periods (Table 1, Fig. 3).

**Table 1:** Results from our Bayesian model on adult female desert bighorn sheep at Lone Mountain, Nevada (2016–2018), with and without offspring

Parameter	Mean	Lower 95% CI	Upper 95% CI	Probability > 0 (%)	Probability < 0 (%)	Overlap with Pre
Pre annual	0.013	− 0.01	0.035	86.00	14.00	–
PP annual	− 0.11	− 0.149	− 0.071	0.00	100.00	0%
AD annual	− 0.02	− 0.082	0.042	26.00	74.00	36%
Pre elevation	− 0.327	− 0.354	− 0.301	0.00	100.00	–
PP elevation	0.373	0.192	0.559	100.00	0.00	0%
AD elevation	− 0.675	− 0.762	− 0.588	0.00	100.00	0%
Pre perennial	0.084	0.066	0.103	100.00	0.00	–
PP perennial	0.303	0.265	0.341	100.00	0.00	0%
AD perennial	0.24	0.188	0.291	100.00	0.00	0%
Pre ruggedness	0.052	0.034	0.069	100.00	0.00	–
PP ruggedness	0.381	0.336	0.429	100.00	0.00	0%
AD ruggedness	0.129	0.081	0.177	100.00	0.00	2%
Pre shrub	− 0.049	− 0.07	− 0.027	0.00	100.00	–
PP shrub	0.501	0.442	0.56	100.00	0.00	0%
AD shrub	0.092	0.026	0.159	100.00	0.00	0%
Pre slope	0.956	0.933	0.978	100.00	0.00	–
PP slope	0.553	0.305	0.762	100.00	0.00	0%
AD slope	0.895	0.821	0.972	100.00	0.00	18%
Pre tree	− 0.906	− 0.95	− 0.862	0.00	100.00	–
PP tree	− 0.414	− 0.634	− 0.201	0.00	100.00	0%
AD tree	− 0.25	− 0.354	− 0.152	0.00	100.00	0%
Pre water	− 2.491	− 2.563	− 2.423	0.00	100.00	–
PP water	− 1.147	− 1.287	− 1.008	0.00	100.00	0%
AD water	− 1.442	− 1.588	− 1.304	0.00	100.00	0%

We report the estimated mean, 95% credible intervals, percent of each credible interval above or below 0, and amount of overlap of each post-parturition category with the non-provisioning category for each habitat parameter. “Pre” indicates habitat parameters for females that were not provisioning offspring (i.e., pre-parturition), “PP” indicates habitat parameters for females that were provisioning offspring, and “AD” indicates habitat parameters for females that had lost their offspring

**Fig. 4:**
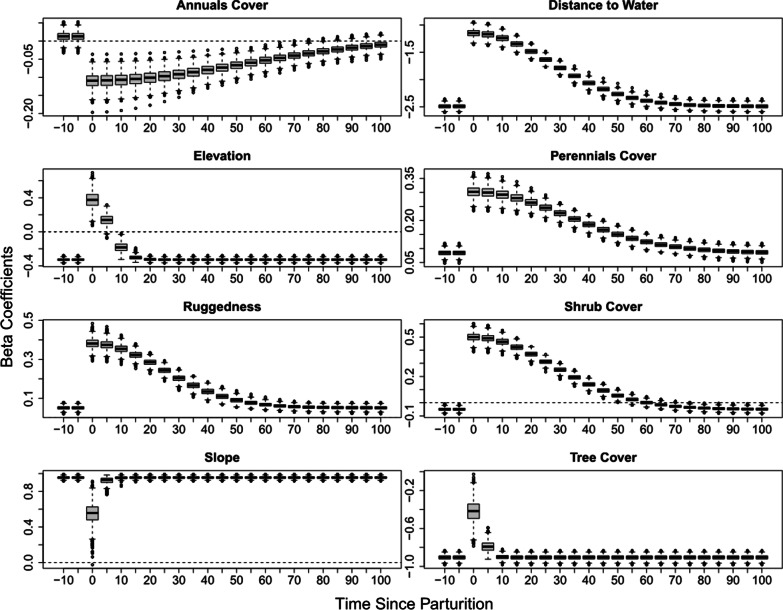
Predictions of habitat variables that contained the post parturition interaction within the top model for habitat selection by desert bighorn sheep at Lone Mountain, Nevada (2016–2018). The y-axis for each pane represents the selection coefficient for each habitat variable and the x-axis represents the days since parturition, with 0 indicating a parturition event

The mean rate of return to pre-parturition levels (e.g., without dependent young) varied across habitat characteristics, ranging from ~ 4 to 108 days (Table 2, Fig. 4). Selection of tree cover, elevation, and slope returned to pre-parturition selection within 2–15 days, while selection of annual grasses and forbs returned to pre-parturition level of selection within 84 to 120 days (Table 2, Fig. 4). The return rates of selection to pre-parturition levels for distance to water, terrain ruggedness, perennial grass and forb cover, and shrub cover all indicate a moderate time to return, from 41 to 75 days (Table 2, Fig. 4).

**Table 2:** Rate of return parameter estimates from our Bayesian model on female desert bighorn sheep at Lone Mountain, Nevada (2016–2018)

Parameter	Mean	Lower 95% CI	Upper 95% CI	Adjusted mean	Adjusted lower 95% CI	Adjusted upper 95% CI
Annuals cover	54.092	42.168	59.808	108.184	84.336	119.616
Elevation	5.720	4.247	7.444	11.440	8.494	14.887
Perennials cover	32.415	27.735	37.734	64.830	55.471	75.468
Ruggedness	24.230	20.526	28.240	48.460	41.053	56.481
Shrub cover	27.353	24.656	30.332	54.706	49.313	60.663
Slope	2.246	1.212	4.104	4.492	2.424	8.207
Tree cover	2.987	2.010	4.181	5.974	4.020	8.362
Distance to water	26.460	24.185	28.865	52.921	48.371	57.729

We present the original estimates from the model and the associated credible intervals, which represent a half-life and not the true return rate, and adjusted parameter estimates that represent the true rate of return for each habitat variable to that of females that were not provisioning offspring. The estimates represent the number of days it takes for each habitat parameter to return to the base level (i.e. females not provisioning offspring)

We estimated selection coefficients for females that lost a neonate to mortality within the first 30 days of life. Several of the selection coefficients observed following the death of a neonate overlapped with selection observed prior to parturition (Table 1, Fig. 3). Following the death of a neonate, females selected low elevations, rugged terrain, steep slopes, and areas close to water (Table 1, Fig. 3). Females that had lost their offspring also selected areas with high shrub cover, moderate annual grass and forb cover, high perennial grass and forb cover, and low tree densities (Table 1, Fig. 3).

## Discussion

Our first objective was to identify the resources and habitat types that are used by female bighorn sheep prior to parturition. We predicted that pre-parturient females would select resources to maximize access to nutritional resources and exhibit more risk-prone behaviors by selecting areas that were less rugged, had shallower slopes, and higher vegetative cover compared to females with dependent young. Pre-parturient females used terrain that was less rugged than females that were provisioning offspring, supporting our hypothesis. Contrary to our hypotheses, however, parturient females selected steeper slopes than when they were provisioning offspring. Selection for less steep slopes, following parturition, may be related to the physical limitations of offspring compared to those of adults, in addition to females already selecting relatively steep slopes prior to parturition for their own safety [20]. Pre-parturient females also selected some areas that were more indicative of a risk-prone strategy, such as selection for lower elevations and areas with higher amounts of annual and perennial grass and forb cover than generally were available. Habitats at lower elevations contained higher presence of coyotes based on camera traps (MEB, unpublished data), in addition to being farther from escape terrain. Therefore, selecting those areas prior to parturition was consistent with females selecting areas to increase their nutritional reserves, which is also consistent with more risk-prone behavior. Contrary to our hypotheses, pre-parturient females selected perennial grass and forb cover to a lesser degree and avoided shrubs compared to females that were provisioning offspring. Those differences are likely attributed to an upward trend in quality of vegetation as the parturition season progressed [18].

Our next objective was to determine if there was a shift in selection of resources immediately following parturition, and if those changes showed a switch from risk-prone to risk-averse strategies. We observed strong support for our prediction that females with dependent young, selected areas that increased safety from predators (e.g., steep slopes and rugged terrain). Following parturition, females adjusted habitat selection to highly rugged areas with less steep slopes and at higher elevations, indicating the importance of areas that provide more safety for offspring. Rugged and steep landscapes are commonly reported as essential habitat types for bighorn sheep and provide females and young with terrain features that increase their ability to evade predators [20, 24, 25, 45, 58]. Immediately following parturition and when provisioning young, females reduced their avoidance of areas with high tree cover, but selected areas with low cover of annual grasses and forbs that were further from water than expected. We expected maternal females to avoid areas with high tree cover because bighorn sheep rely on vision to detect approaching predators [67, 68]. Trees provide additional cover for stalking predators, such as mountain lions [38, 69] that were more abundant at mid- to high-elevations throughout the study area (MEB, unpublished data). The reduced avoidance of this habitat type likely is related to the increase of tree densities at higher elevations, which females moved to following parturition.

Our models generally supported the prediction that females that lost offspring shifted to areas similar to those selected prior to parturition, although the credible intervals following loss of a neonate were more variable than those of pre-parturient females. The only strong differences between pre-parturient females and those that lost their young were selection for areas close to water and areas with greater tree cover. Females without young were less likely to avoid high tree cover, perhaps because they could be more proficient at avoiding predators than if they had a dependent offspring. Instead of moving close to sources of water similar to pre-parturient females, those that lost offspring remained farther than expected from water, similar to individuals that were provisioning offspring. Desert bighorn sheep may not start using water sources heavily until the onset of summer [70–72]; females that lost offspring were not constrained by availability of surface water during April and May, when water needs can be satisfied by preformed water in vegetation [73, 74]. Overall, females that lost their offspring mostly shifted to similar levels of selection exhibited by pre-parturient females, supporting the prediction that individuals partake in tradeoffs based on provisioning status.

Finally, our results somewhat supported the hypothesis that females shifted from areas associated with risk-averse behaviors having a focus on safety of young (i.e., steep slopes and rugged and open terrain) to sites associated with risk-prone behaviors with a focus on nutritional resources (i.e., vegetation cover, shallower slopes, and less rugged terrain) within one month following parturition because older offspring were at a reduced risk of predation. Females provisioning offspring quickly returned to selecting areas with lower elevations, steeper slopes, and lower tree cover that were similar to those selected by females not provisioning offspring. Those shifts occurred from 4 to 15 days following parturition and are likely explained by the need for high quality nutrition during lactation. Lower elevations contained more annual and perennial grasses and forbs, which are important sources of digestible energy and protein during lactation [20, 75–77]. Lower elevations also were associated with greater abundance of coyotes, but females still selected areas with rugged terrain and steep slopes, which are beneficial for avoiding coursing predators (MEB, unpublished data [20, 78, 79]). The rapid return to steeper slopes likely is related to how quickly bighorn sheep neonates become proficient at maneuvering in these rugged landscapes. During captures, neonates that were greater than two days old were highly mobile and moved very effectively through precipitous terrain (MEB, personal observation); therefore, it is likely that females moved into steeper areas shortly after birth to further increase safety for neonates.

Females with dependent young took much longer to return to non-provisioning levels of selection of other habitat types (i.e., annual grasses and forbs, perennial grasses and forbs, shrub cover, and rugged terrain). Females resided in more rugged terrain, with greater shrub cover and further from water for up to 2.5 months following parturition. This slow return to selection for habitat that was similar to non-provisioning levels likely is the result of females preferring the extra security for juveniles in these areas. Adjustments to selection for elevation may have increased nutrient acquisition and allowed individuals to still select habitat types that provided extra security for neonates, resulting in prolonged rates of return for variables such as rugged terrain and shrub cover. We suspect that the slow return to selection for areas close to water was linked initially to slowly rising temperatures as summer progressed in this arid region [70, 74]. About 2 months following the mean date of parturition, which was April 12 in our study, temperatures increased dramatically, resulting in more frequent visits to water, which is common in areas occupied by desert bighorn sheep during summer months compared to other times of year [20, 70–72]. Females may have slowly decreased use of areas with high shrub cover to take advantage of the digestible energy and protein associated with species such as Stansbury cliffrose [80]. Selection for these habitats likely allowed females to access sufficient nutrition, while also moving closer to water to account for decreasing water content in vegetation [74], and simultaneously selecting habitat types that provide security for themselves and offspring. Diet content and quality of this population of bighorn sheep indicated that females provisioning offspring had high concentrations of shrubs in their diet, while also being more selective of forage species when compared to diets prior to parturition [18].

Animals typically select habitats that increase their access to high-quality forage and positively influence their reproductive fitness. Habitat selection often varies throughout the year and throughout the lifetime of an individual [1, 81, 82]. This variation may be in response to seasonal changes in availability of resources in their home range, climate, or stem from changes in reproductive status [9, 20, 37, 83]. We expected individuals to adjust how they select habitats following parturition. Females provisioning young should select habitats with high quality forage, because of the high nutritional demands of lactation [34, 77], that is proximate to or within areas that provide safety for offspring. Those ideal conditions of high safety and high-quality forages rarely exist simultaneously [28]; consequently, individuals often are forced to make tradeoffs in selection of resources with changes in their reproductive state to maximize their reproductive fitness. Rugged landscapes with access to steep slopes, areas commonly occupied by montane ungulates, may have limited amounts of forage that will meet the nutritional demands of lactation. Females likely must trade access to high quality vegetation for safety of their young, but eventually lessen costs associated with this tradeoff to increase nutrient acquisition. We predicted that relationship would be a product of the degree of tradeoff made by females and the ability of offspring to avoid predation.

Areas that provided both high quality habitats for safety of offspring and for nutritional resources for maternal females were rare throughout the study area (Additional file 1 and 2). For instance, slopes greater than 50° rarely contained high concentrations of grass and forb cover. High amounts of shrub cover also were less prevalent in the steepest and most rugged terrain. Our results suggest that females were making a tradeoff between survival of offspring and availability of nutritional resources when provisioning offspring, because some risk-averse strategies were relaxed when females were not provisioning offspring. Indeed, females that lost a neonate, or that were not pregnant, had higher levels of fecal nitrogen than those individuals that were provisioning offspring [18].

Our results were generally consistent with other studies that investigated tradeoffs in habitat selection between females provisioning offspring and those that were not. For instance, Bleich et al. [20] found that females with offspring selected more rugged terrain and steeper slopes than those without offspring. Barten et al. [21] observed shifts, by female caribou, in use of elevation that was linked with survival of young. Similarly, selection for high elevations differed between females provisioning and those not provisioning offspring in our study. Heffelfinger et al. [17], however, reported little support for mule deer trading nutritional quality for areas that increased safety of offspring, but habitats that provided both safety for the offspring and nutrition for the mother were present in their study area. Nevertheless, we observed clear shifts in how bighorn sheep selected those resources with reproductive status. When combined with diet results from Blum et al. [18], we demonstrated clear tradeoffs in habitat selection relative to provisioning status.

Similar tradeoffs have been hypothesized and demonstrated in other species of large mammals [4, 17, 23, 25, 28, 39, 42, 84, 85]. As with bighorn sheep, we expect similar responses from other mountain ungulates, especially other sheep species, that utilize precipitous terrain to reduce the likelihood of predation on neonates [21, 28, 43]. However, we expect the nature of these tradeoffs to vary somewhat across ecosystems and species. For instance, the return rate of each variable is likely dependent on the mobility of neonates, nutritional condition of maternal females, habitat types that decrease predation risk available to individuals, and nutritional availability across the landscape. Predator avoidance by neonates and age-dependent mobility vary across species and should influence how quickly female ungulates can utilize areas that are more risky, but more nutritionally productive [86, 87]. Furthermore, the number of neonates that a mother has may also influence length of the tradeoff. Bighorn sheep give birth to a single offspring, whereas species such as mule deer, moose (*Alces alces*), and several Asiatic species of sheep more commonly give birth to multiple offspring [39, 88–90]. We expect the length of the tradeoff to be shorter in species that have multiple offspring, because nutritional condition of females depreciates faster with more mouths to feed. However, this effect may also be mitigated by increasing time spent in areas with abundant high-quality forage prior to parturition, to increase somatic reserves [77].

While the length of tradeoffs likely varies between ecosystems and species, behaviors such as returning to pre-parturition selection following the loss of a neonate will likely occur in all ecosystems. Our study showed that females rapidly reverted to pre-parturition levels of habitat selection following the loss of a neonate, providing evidence that female ungulates will shift their focus to increasing future reproductive potential. Females that have lost a neonate no longer can increase their fitness through survival of that offspring; as a result of that loss, however, they are able to replenish somatic reserves expended during gestation, thereby preparing for the next breeding season and potentially enhancing survival of their next offspring [18, 34, 77]. Females in better nutritional condition have higher pregnancy rates and give birth to larger offspring that have higher survival rates [15, 30, 52, 91, 92]. Therefore, females that shift to areas with increased access to high-quality forage are likely enhancing their fitness by increasing the likelihood that future offspring survives.

## Conclusions

Individuals exhibit tradeoffs in selection associated with predation risk and nutritional acquisition, both of which enhance their fitness. Those differences in selection among reproductive states must be accounted for in habitat models as well as conservation efforts to ensure that models of habitat selection properly identify population-level effects [93, 94]. Further, identifying those tradeoffs in wild populations provides insight into the evolutionary processes that have shaped the life histories of bighorn sheep and other montane ungulates [28, 43, 95, 96]. Our results provide valuable insight into changes in selection of resources relative to reproductive status and rearing of offspring, which are essential to sustaining viable populations on the landscape [93, 97, 98]. Our results also demonstrate the importance of accounting for differences in habitat selection between parturient and non-parturient females when developing habitat models. Habitat models that do not account for these different strategies may result in less predictive species distribution models. Accounting for that variation in selection likely will identify areas that are important for conservation that, otherwise, may be overlooked.

## Supplementary Information

**Additional file 1.** Methodology and results from linear regression between risk-prone and risk-averse habitat covariates.

**Additional file 2: Figure 1.** Scatter plot of raw data points from risk-prone and risk-averse habitat covariates on Lone Mountain, Nevada (2016–2018). The y-axis represents risk-prone habitat covariates, and the x-axis represents risk-averse habitat covariates. Each row has the same y-axis covariate, and each column has the same x-axis covariate. Regression lines and the associated R-squared values are plotted for each relationship.

## Data Availability

The datasets generated for this study are available on request to the corresponding author.
