# Correction to: Changes in selection of resources with reproductive state in a montane ungulate

**DOI:** 10.1186/s40462-023-00399-w

**Published:** 2023-07-05

**Authors:** Marcus E. Blum, Kelley M. Stewart, Kevin T. Shoemaker, Mike Cox, Brian F. Wakeling, Thomas E. Dilts, Joe R. Bennett, Vernon C. Bleich

**Affiliations:** 1 Natural Resources Institute, Texas A&M University, 1001 Holleman Dr College Station, 77840 Texas, USA; 2 Department of Natural Resources and Environmental Science, University of Nevada, Reno 1664 N. Virginia St., MS 186, 89557 Reno, NV USA; 3 Nevada Department of Wildlife, 6980 Sierra Center Parkway #120, 89511 Reno, NV USA; 4 Montana Fish, Wildlife, and Parks, P.O. Box 200701, 59620 Helena, MT USA

**Correction to:** ***Movement Ecology (2023) 11:20***

10.1186/s40462-023-00378-1

Following publication of the original article [1], the authors reported incorrect information in the ‘Availability of data and materials’ section.

The statement in the ‘Availability of data and materials’ section originally read: We will make our data publicly available upon publication.

The statement should read: The datasets generated for this study are available on request to the corresponding author.

The original article [1] has been updated.
