# Supplementary material for: Changes in selection of resources with reproductive state in a montane ungulate
Source: Mov Ecol. 2023 Apr 5;11:20. doi: 10.1186/s40462-023-00378-1 (PMC10077753; doi:10.1186/s40462-023-00378-1)
Supplement: Supplementary file 1 — Additional file 1. Methodology and results from linear regression between risk-prone and risk-averse habitat covariates. [file 40462_2023_378_MOESM1_ESM.docx]

**Supplementary Material 1**

High levels of vegetation cover may occur within rugged terrain and on steep slopes, removing the need to tradeoff resources. We defined availability of habitat types associated with risk-averse (i.e., high amounts of rugged terrain and steep slopes) and risk-prone (i.e., high vegetation cover) behavior by using the raster layers of various resources in our study area to determine if resources associated with different risk strategies coexisted. We generated points in the center of each raster pixel across the study area and extracted the associated terrain ruggedness, slope, shrub, annual grass and forb, and perennial grass and forb cover values to those locations. All covariates were standardized prior to analysis. We then conducted linear regressions, in a frequentist framework, between each risk-averse (terrain ruggedness and slope) and risk-prone (shrub, annual grass and forb, and perennial grass and forb cover) habitat covariate across the entire study area to investigate relationships between each risk-averse and risk-prone variable. To determine if associations existed between different values of habitat variables, we calculated trend lines and R^2^ values for each regression between risk-averse and risk-prone habitat types.

All relationships between risk-prone and risk-averse habitat types were significant, however, none of these relationships were strong (Table 1, Figure 1). Only the regressions between annual grass and forb cover, slope, and terrain ruggedness resulted in a negative relationship, indicating that as terrain ruggedness increased, annual grass and forb cover decreased. The remaining positive relationships were weak and there were noticeable declines in availability of higher vegetation cover at slopes greater than 50 degrees (Figure 1). Similarly, as terrain became more rugged there were fewer areas with high amounts of shrub, annual, or perennial grass and forb cover. Based on these results, female bighorn sheep did not appear to have access to both high quality risk-averse and risk-prone habitat types in the same areas.

Table 1. Results from linear regression between risk-prone and risk-averse habitat covariates on Lone Mountain, Nevada (2016-2018).

| **Risk-Prone Variable** | **Risk-Averse Variable** | **Beta Coefficient** | **Lower 95% CI** | **Upper 95% CI** | **R-Squared** |
| --- | --- | --- | --- | --- | --- |
| Shrub Cover | Slope Degree | 0.113 | 0.111 | 0.115 | 0.013 |
| Shrub Cover | Ruggedness | 0.047 | 0.045 | 0.049 | 0.002 |
| Annuals Cover | Slope Degree | -0.136 | -0.138 | -0.134 | 0.019 |
| Annuals Cover | Ruggedness | -0.087 | -0.089 | -0.085 | 0.008 |
| Perennials Cover | Slope Degree | 0.108 | 0.106 | 0.11 | 0.012 |
| Perennials Cover | Ruggedness | 0.066 | 0.064 | 0.068 | 0.004 |

Figure 1. Scatter plot of raw data points from risk-prone and risk-averse habitat covariates on Lone Mountain, Nevada (2016-2018). The y-axis represents risk-prone habitat covariates, and the x-axis represents risk-averse habitat covariates. Each row has the same y-axis covariate, and each column has the same x-axis covariate. Regression lines and the associated R-squared values are plotted for each relationship.
