# Supplementary figures and images for: Changes in selection of resources with reproductive state in a montane ungulate
Source: Mov Ecol. 2023 Apr 5;11:20. doi: 10.1186/s40462-023-00378-1 (PMC10077753; doi:10.1186/s40462-023-00378-1)

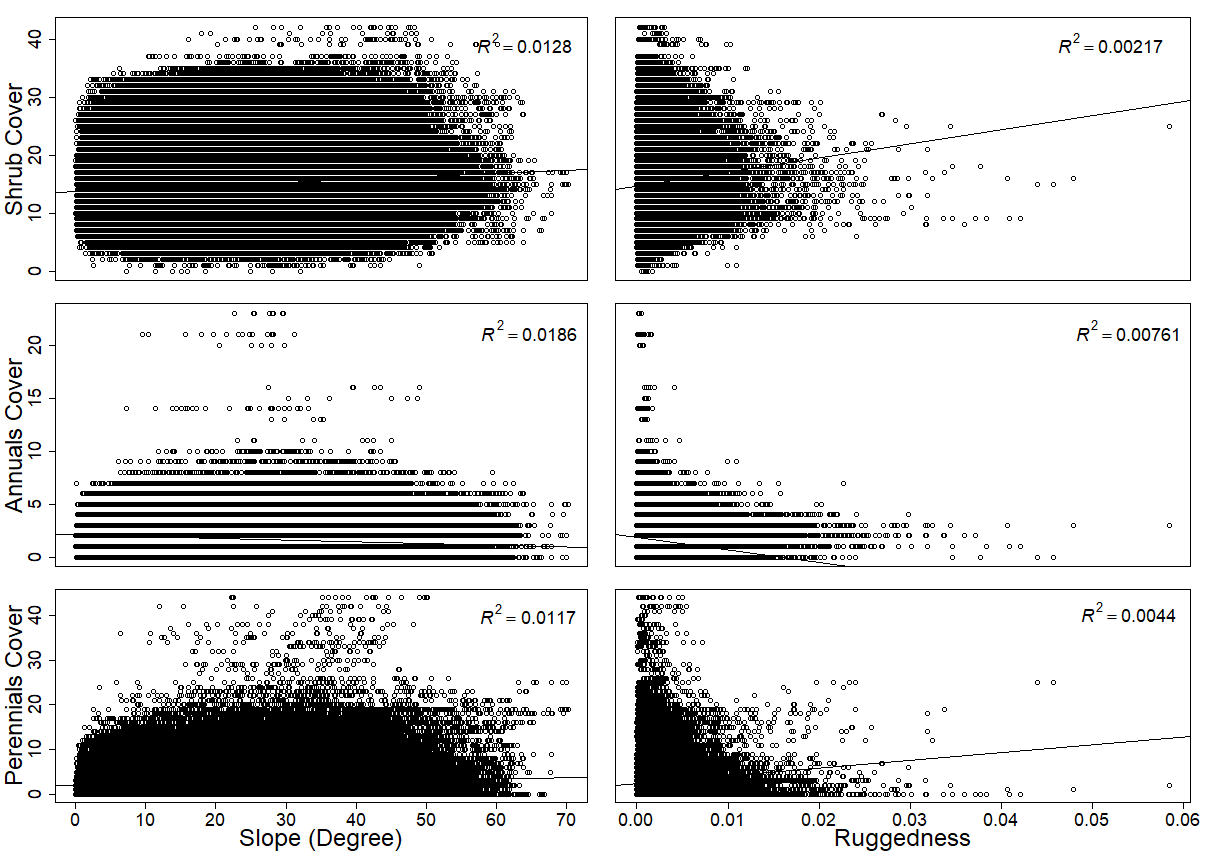

Supplement: Supplementary file 2 — Additional file 2: Figure 1. Scatter plot of raw data points from risk-prone and risk-averse habitat covariates on Lone Mountain, Nevada (2016–2018). The y-axis represents risk-prone habitat covariates, and the x-axis represents risk-averse habitat covariates. Each row has the same y-axis covariate, and each column has the same x-axis covariate. Regression lines and the associated R-squared values are plotted for each relationship. [file 40462_2023_378_MOESM2_ESM.png]
